# Biological Control of *Escherichia coli* O157:H7 in Dairy Manure-Based Compost Using Competitive Exclusion Microorganisms

**DOI:** 10.3390/pathogens13050361

**Published:** 2024-04-27

**Authors:** Xiuping Jiang, Jingxue Wang

**Affiliations:** 1 Department of Food, Nutrition, and Packaging Sciences, Clemson University, Clemson, SC 29634, USA; 2 Department of Food Science and Engineering, Ocean University of China, Qingdao 266003, China; snow@ouc.edu.cn

**Keywords:** compost safety, *Escherichia coli* O157:H7, competitive exclusion microorganism, growth inhibition, biological soil amendment

## Abstract

Background: Animal manure-based compost is a valuable organic fertilizer and biological soil amendment. To ensure the microbiological safety of compost products, the effectiveness of competitive exclusion microorganisms (CE) in reducing *Escherichia coli* O157:H7 in dairy manure-based compost was evaluated. Methods: A cocktail of *E. coli* O157:H7 strains were inoculated into dairy compost along with CE strains isolated from compost, and the reduction in *E. coli* O157:H7 by CE was determined in compost with 20%, 30%, and 40% moisture levels at 22 °C and 30 °C under laboratory conditions, as well as in fall, winter, and summer seasons under greenhouse settings. Results: Under lab conditions, CE addition resulted in 1.1–3.36 log reductions in *E. coli* O157:H7 in compost, with enhanced pathogen reduction by higher moisture and lower temperature. In the greenhouse, >99% of the *E. coli* O157:H7 population in compost with ≥30% moisture due to cross-contamination can be effectively inactivated by CE within 2 days during colder seasons. However, it took ≥8 days to achieve the same level of reduction for heat-adapted *E. coli* O157:H7 cells. Conclusions: Our results demonstrated that the competitive exclusion of microorganisms can be an effective tool for controlling foodborne pathogens in compost and reducing the potential for soil and crop contamination.

## 1. Introduction

*Escherichia coli* O157:H7, a leading foodborne pathogen, is commonly shed in the feces of cattle and other food-producing animals. Numerous studies have reported the prolonged survival of *E. coli* O157:H7 in raw manure, thereby heightening the risk of its transmission into the food chain and posing a public health threat [1,2]. Indeed, outbreaks of *E. coli* O157:H7 infections have frequently been linked to the consumption of fresh produce or other food products directly or indirectly contaminated by water or manure containing this foodborne pathogen [3].

Due to the presence of human pathogens in raw animal wastes, the proper composting of these wastes and handling of the finished products are critical for ensuring the safety of fresh produce production when the animal manure-based compost is used as a fertilizer and biological soil amendment. Importantly, the Food and Drug Administration’s (FDA) Food Safety Modernization Act (FSMA) Produce Safety Rule has placed limitations on the use of raw manure and has also established microbial standards for composted manure used on crops produced for direct human consumption [4].

Composting is an aerobic process during which organic waste is biologically degraded by microorganisms to humus-like material. Both bacteria and fungi are present and active in a typical composting process [5,6,7]. Most of the foodborne pathogens inherently present in the raw manure are inactivated during the thermophilic phase due to high temperature [5,8]. Furthermore, compost contains a wealth of microbial species; however, these organisms face fierce competition within their environment. Compost microorganisms can interact synergistically or compete for the available nutrients [9,10]. In this complex ecosystem, it is likely that some microorganisms have acquired protective features, such as the secretion of biocidal compounds. Bacterial competition in the environment can be classified as exploitative competition, where bacteria utilize limited nutrients or compete for colonizing sites, thereby depriving fellow microorganisms of the same genotypes, and interference competition, where cell damage occurs via the release of bioactive compounds by other microorganisms [10,11]. As a result, certain populations of compost microflora may possess antimicrobial activities against harmful human pathogens.

Biocontrol of foodborne pathogens in agricultural settings, such as animal production, fresh produce fields, and food processing environments, has been reported [11,12,13,14]. This approach seems feasible since these microorganisms originated from agricultural environments and would be adapted to their native environment. Another advantage is that, ultimately, the usage of biocontrol agents against foodborne pathogens leads to less reliance on harmful chemicals and sanitizers by the food industry.

The objective of this study was to isolate microorganisms from compost samples that produce metabolites bacteriostatic or bactericidal to *E. coli* O157:H7 and then determine their ability to inhibit the growth of the pathogen in dairy compost under laboratory and greenhouse conditions.

## 2. Materials and Methods

### 2.1. Bacterial Strains and Culture Conditions

Due to strain variation in growth parameters and persistence, a cocktail of three to five *E. coli* O157:H7 strains was used for this study. Five *E. coli* O157:H7 strains (spinach outbreak strain F06M-0923-21 and Taco John’s outbreak strain F07M-020-1, both obtained from California Department of Health [15], avirulent strain B6914 *stx* 1^−^ and 2^−^ obtained from Dr. Pina Fratamico, USDA-ARS-ERRC [16], and avirulent strains MD46 and MD47 obtained from Dr. Mike Doyle at the University of Georgia) were used in this study [17]. To differentiate from the competitive exclusion (CE) strains or the compost microflora, all tested *E. coli* O157:H7 strains were induced to be rifampicin-resistant via the gradient plate method [18], and no antagonistic effect was observed among these strains. Prior to each experiment, the strains from the freezer stocks were streaked on Tryptic Soy Agar supplemented with 100 µg mL^−1^ rifampicin (Fisher Scientific, Fair Lawn, NJ, USA) (TSA-R) plates and incubated at 35 °C for 24 h. Single colonies were inoculated into Tryptic Soy Broth (TSB) without glucose, grown to an early stationary phase, and used in further experiments.

### 2.2. Competitive Exclusion Microorganism Isolation and Culture Conditions

The CE strains were isolated from 31 samples of finished composts, including poultry litter-, dairy manure- and plant wastes-based as described previously [19]. Briefly, 9 mL of universal pre-enrichment broth (UPB) was added to each compost sample (ca. 1 g), and the mixtures were serially diluted (1:10) in phosphate-buffered saline (PBS). A volume of 0.1 mL of each dilution was plated in duplicate on tryptone, yeast extract, proteose peptone 3 agar plates (TYP) containing proteose peptone 3 (5 g L^−1^), tryptone (5 g L^−1^), yeast extract (5 g L^−1^), sodium chloride (8 g L^−1^), and agar (17 g L^−1^) and incubated at room temperature. The colonies were randomly selected from plates and streaked several times for isolation.

Two methods, including a spot-on-lawn assay and liquid co-culture experiments, were used to screen isolates for antimicrobial activity against *E. coli* O157:H7 strains. In addition to selection at room temperature, some isolates were tested for antimicrobial activity at 42 °C. For the spot-on-lawn assay, 0.1 mL of approximately 10^7^ CFU ml^−1^ cells of the 3-strain cocktail of *E. coli* O157:H7 (F06M-0923-21, F07M-020-1, and B6914) were plated in duplicate onto the surface of TYP plate. Putative CE isolates were grown individually on TYP plates at 25 °C for 48 h; then, a single colony was replica-plated on a sterile TYP plate and a TYP plate containing *E. coli* O157:H7 strains as the indicator microorganism. The plates were incubated at 25 °C for 48 h and then observed for zones of inhibition.

The CE isolates were selected for a liquid co-culture experiment based on their antimicrobial activity against *E. coli* O157:H7 expressed as a clear inhibition zone. For the liquid co-culture experiments, *E. coli* O157:H7 strain B6914 was grown to stationary phase at room temperature on a rotary shaker in TYP broth. The putative CE isolates were grown in similar conditions. To test the inhibitory capacity in TYP broth, CE isolates were inoculated in equal concentration (ca. 10^2^ CFU/mL) with the target *E. coli* O157:H7. Concurrently, individual CE strains and *E. coli* O157:H7 strain were inoculated in TYP broth separately and monitored for growth. Samples were collected at selected intervals and plated on TSA-R to enumerate only *E. coli* O157:H7 or TSA for CE isolates.

### 2.3. Species Identification by Amplifying the 16S rRNA Gene

The DNA of potential CE isolates from the compost samples was extracted using the UltraClean^TM^ Microbial DNA Isolation Kit (Mo-Bio Laboratories, Inc., Carlsbad, CA, USA) as described in the manufacturer’s instructions. Isolates were identified by PCR amplification of 16S rRNA genes using universal primers and sequenced by Eurofins Genomics (Louisville, KY, USA) as described previously [19]. The forward primer ENV1 (5′-AGA GTT TGA TII TGG CTC AG-3′) targets positions 8–27 of *E. coli* 16S rRNA, whereas the reverse primer ENV2 (5′-CGG ITA CCT TGT TAC GAC TT-30′) corresponds to positions 1511–1492 [20]. PCR reagents were used as a negative control, while the *E. coli* O157:H7 DNA was used as a positive control. The bacterial species was identified using BLAST (NSBI) and The Ribosomal Database Program [19].

### 2.4. Compost Inoculation, Sampling and Bacterial Enumeration

Finished dairy waste—based compost (Black Kow^®^, Black Gold Compost Co., Oxford, FL, USA) was used to determine the efficacy of CE strains against *E. coli* O157:H7 under both laboratory and greenhouse conditions. Prior to experiments, large particles present in compost samples were removed by sieving (sieve pore size, 0.3 × 0.3 cm). Compost was placed in sterile containers under refrigeration conditions and used for further experiments.

#### 2.4.1. *E. coli* O157:H7 Growth under Laboratory Conditions

The selected CE strains (*n* = 3) were grown in TSB without glucose to the early stationary phase and then centrifuged and washed twice with 0.8% saline solution. To determine the effectiveness of CE on *E. coli* O157 inhibition in the compost, about 4 logs of the 3-strain cocktail of CE cultures were inoculated into the above compost containing ca. 6 logs of indigenous microorganisms using the spraying method [19]. The CE-inoculated compost was then adjusted with sterile tap water to different moisture contents (20, 30, and 40%) and then acclimated at room temperature for 24 h. The overnight cultures of three rifampicin-resistant *E. coli* O157:H7 strains (F06M-0923-21, F07M-020-1, and B6914) grown in TSB-R broth were washed with saline and then inoculated to the CE-inoculated compost at an initial concentration of ca. 2 log CFU/g, and the inoculated samples were then stored at temperatures of 22 or 30 °C. At selected intervals, compost samples were enumerated for *E. coli* O157:H7 on TSA-R plates.

#### 2.4.2. *E. coli* O157:H7 Growth under Greenhouse Conditions

Two experimental approaches were conducted in the greenhouse. Both CE strains and *E. coli* O157:H7 strains were prepared as described above. The first approach was to simulate pathogen contamination of the finished compost. Briefly, the finished compost with adjusted moisture levels of 20, 30, and 40% were first inoculated (at a ratio of 1:10 v/wt) with the 10-strain cocktail of CE cultures to reach ca. 10^8^–10^9^ CFU g^−1^. After 24 h, the compost was inoculated with a cocktail of three avirulent *E. coli* O157:H7 strains (B6914, MD46, and MD47) at ca. 10^5^–10^6^ CFU g^−1^. Samples consisted of (i) compost inoculated only with *E. coli* O157:H7 cocktail, (ii) compost inoculated only with CE cocktail, (iii) compost inoculated with both *E. coli* O157:H7 and CE cocktail, and (iv) uninoculated compost.

The second approach was to simulate the survival of the pathogen during thermophilic composting. To prepare for heat-adapted cells in compost, above-avirulent *E. coli* O157:H7 cocktail strains were inoculated (1:10 v/wt) to the finished compost with 40% MC, subjected to heat at 48 °C for 30 min and then inoculated further at a ratio of 1:10 wt/wt in compost samples with 40, 30, and 20% MC. After 24 h incubation at room temperature, the *E. coli* O157:H7 inoculated compost samples were inoculated (1:10 v/wt) with the 10-strain cocktail of CE cultures to reach ca.10^8^–10^9^ CFU g^−1^. Four treatments of compost samples were prepared the same as described in the first approach.

For both approaches, two independent experiments were performed in triplicate. Experiments were performed as follows: Summer trials (August–September), Fall trials (October–December), and Winter trials (February–March) inside a greenhouse. Sterile cups containing compost samples were arranged in large plastic containers, and a digital hydrothermometer (EU 620-0915; VWR International, Radnor, PA, USA) for temperature and relative humidity was placed inside. Containers had recipients with saturated KCl solution and were closed every evening and opened in the morning. The moisture levels of the samples were adjusted every evening based on weight loss. Adjustment in the morning was not necessary since there was little moisture loss due to the overnight storage in high relative humidity. Therefore, samples were subjected to lower temperatures and high relative humidity overnight and high temperatures and decreased humidity during the day.

Treatments were sampled on day 2 then every 4 days and analyzed for moisture content (the moisture levels of the samples were adjusted every day in the greenhouse for all samples) and bacterial enumeration. Briefly, 5 g of inoculated compost was mixed and homogenized with 45 mL of PBS in a sterile stomacher bag. The samples were then serially diluted and plated on TSA-R for the enumeration of *E. coli* O157:H7 or TSA for the enumeration of CE or the compost microflora. Data obtained from bacterial enumeration were expressed as log CFU per gram dry weight (CFU g/dw), and the detection limit of the plating method was approximately 100 CFU g/dw [19].

### 2.5. Statistical Analysis

The analysis of pathogen survival data was performed using JMP 11.2.1 (SAS Institute Inc., Atlanta, GA, USA). Analysis of variance (ANOVA), followed by the least significant differences (LSD) test, was carried out to determine whether significant differences (*p* < 0.05) existed among different treatments.

## 3. Results

### 3.1. Isolation and Identification of CE Bacteria against E. coli O157:H7

Potential CE microorganisms were isolated from various samples, including dairy manure-based and chicken litter-based finished compost, plant-based compost, and commercial organic fertilizers (*n* = 31). The 786 phenotypically different colonies were purified and tested for inhibition activity against *E. coli* O157:H7 using the spot-on-lawn method followed by broth co-culture method. A total of 22 isolates were considered as potential CE microorganisms. In the presence of individual CE strains, *E. coli* O157:H7 population reduction ranged from 1.1 to 3.9 logs in TYP broth and 0.9 to 3.7 logs in compost, with *Kluyvera* strain as the most effective (Table S1). These CE isolates were identified as *Brevibacillus parabrevis*, *Bacillus amyloliquefaciens*, *Pseudomonas thermotolerans*, *Comamonas testosterone*, *Enterobacter*, *Citrobacter*, *Raoultella*, *Kluyvera*, unclassified *Comanondaceae*, and unclassified *Enterobacteriaceae* by 16S rRNA method. Three CE isolates (*B. parabrevis*, *B. amyloliquefaciens,* and *P. thermotolerans*) were selected for laboratory trials, and ten CE isolates (Table S1) were used for the greenhouse study.

### 3.2. Effectiveness of CE Treatment on the Growth Reduction in E. coli O157:H7 in Compost under Laboratory Conditions

Under laboratory conditions, *E. coli* O157:H7 grew in the compost with or without CE application under three moisture levels (20, 30, and 40%) and two temperatures (22 and 30 °C) (Table 1 and Table S2). As compared with the controls, the CE treatment was effective by reducing the growth of *E. coli* O157 within 3 days of incubation at 22 and 30 °C by 1.1~2.1, 2.2 ~2.6, and 2.6~3.4 logs in compost with moisture levels of 20, 30, and 40%, respectively. For the compost with 20% moisture, there was more reduction in *E. coli* O157:H7 at 30 °C than at 22 °C; however, at higher moisture contents (30 and 40%), CE reduced slightly more *E. coli* O157:H7 population at a lower temperature (22 °C).

### 3.3. Effectiveness of CE Treatment on the Growth Reduction in E. coli O157:H7 in Compost under Greenhouse Conditions

**Figure 1:**
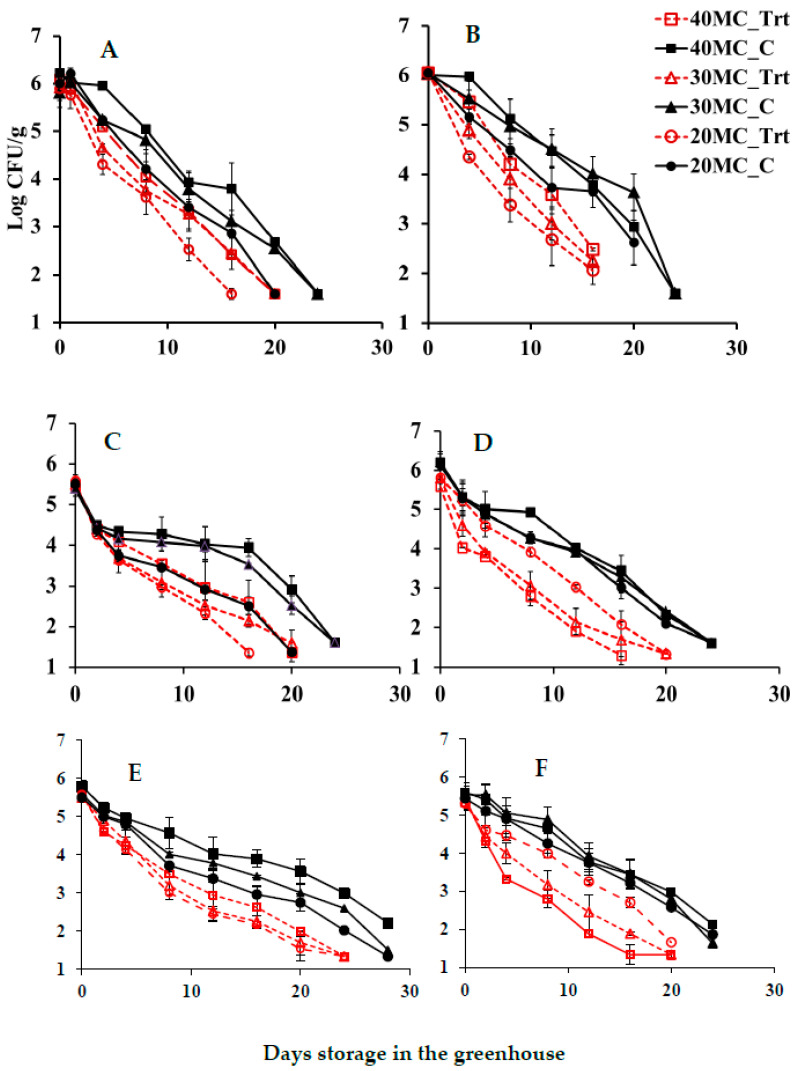
Inhibition of *E. coli* O157:H7 in the presence of CE in the greenhouse conditions. (**A**) Summer trial heat-adapted cells; (**B**) Summer trial non-adopted cells: (**C**) Fall trial heat-adapted; (**D**) Fall trial non-adapted; (**E**) Winter trial heat-adapted and (**F**) Winter trial non-adapted cells.

To test the effects of seasonal changes on bacterial inactivation, experiments were performed in the fall, winter, and summer seasons. The average values of temperature in the greenhouse were 24.4, 21.2, and 28.4 °C for fall, winter, and summer trials, respectively, while the average values of relative humidity in the greenhouse were 42.9, 28.0, and 55.4%, respectively. Two different scenarios for pathogen inoculation were tested: a possible recontamination event of the finished compost and the presence of heat-adapted cells that survived the thermophilic phase of composting. For the controls, the season or the compost moisture levels did not influence overall the pathogen survival in the compost samples (Figure 1). In the presence of CE microorganisms, *E. coli* O157:H7 inoculated in composts with high moisture levels (30 and 40%) declined faster than in the compost with low moisture levels (20% MC) regardless of the inoculation method. Overall, the *E. coli* O157:H7 population was reduced more for non-adapted cells (0.06 to 2.14 log CFU/g) than the heat-adapted cells (0.02 to 1.54 log CFU/g) by CE treatment for all trials. These results demonstrated the impact of bacterial physiological state and moisture levels on pathogen survival in the compost environment.

Seasons influenced the rate of pathogen inactivation. Although *E. coli* O157:H7 declined in CE-treated samples in all cases as compared with the controls, significant inactivation of non-adapted *E. coli* O157:H7 by CE microorganisms occurred after only 2 days of storage in the greenhouse in compost samples with higher moisture content (40 and 30%) during the fall and winter trials (Table 2). In the compost with 20% MC, a significant reduction in *E. coli* O157:H7 by CE microorganisms took 16 days of storage for the same conditions. On the other hand, the heat-adapted cells showed resistance to inhibitory action by CE since significant differences between treatments and controls were present after 12 days for compost with 40 and 30% MC and 16 days of storage for compost with 20% MC in the fall trial. A similar outcome resulted from the winter trial: heat-adapted cells with CE treatments showed differences compared to controls in compost with 40% moisture content at day 8, 30% moisture content at day 12, and 20% moisture content at day 16 of greenhouse storage. As for the summer trial, there was no significant difference between the treatment and the controls in the first 4 days of greenhouse incubation for both heat-adapted and non-heat-adapted cells. *E. coli* O157:H7 population in most of the treatments dropped to significant levels after 8 days of storage in the greenhouse. The temperature in the greenhouse varied greatly between the three tested seasons (in the summer trial, occasional temperatures over 50 °C were recorded in the sample, whereas in the fall and winter, the temperature did not exceed 38 °C). Also, some of the CE strains did not grow at elevated temperatures (42 °C) and therefore may be less active when exposed to elevated temperatures.

## 4. Discussion

Composting is an environmentally friendly process for converting livestock and agricultural wastes into organic fertilizer and soil amendment. During composting, the high temperatures achieved in the thermophilic phase are critical for pathogen inactivation. However, despite high temperatures, extended survival of pathogens in compost has been reported [12,21]. This study evaluated the effectiveness of selected competitive exclusion microorganisms (CE) isolated from composts for inactivating *E. coli* O157:H7 in dairy compost with different moisture levels under both laboratory and greenhouse conditions.

According to the literature, lactic acid bacteria, *Enterococcus*, *Pseudomonas*, *Paenibacillus*, *Streptomyces*, *Bacillus*, and some commercially produced bacterial cultures have been widely used as CE microorganisms in controlling foodborne pathogens [22,23,24]. Some of the CE species identified in this study were previously reported as possessing inhibitory activities against both human and plant pathogens. For example, *Pseudomonas aeruginosa* ISO1 and ISO2 isolated from the compost inhibited plant pathogens *Pythium aphanidermatum* and *Fusarium solani* [25]. Wang and Jiang [19] reported the inhibition of 10 fresh-produce outbreak strains of *Listeria monocytogenes* up to 2.2 logs by 17 CE strains isolated from compost, including *Bacillus* spp., *Brevibacillus* spp., *Kocuria* spp., *Paenibacillus* spp., and *Planococcus* spp. Additionally, *Kluyvera*, a soil bacterium, exhibited a significant reduction in *E. coli* O157:H7 in both liquid broth and compost (Table S1). A previous study reported that *Kluyvera ascorbate* SUD165 could protect canola, Indian mustard, and tomato seedlings against the inhibitory effects of high concentrations of heavy metals such as nickel, lead, and zinc by providing the plants with sufficient ions [26]. Iron is an essential micronutrient for most pathogenic bacteria, including *E. coli* O157:H7, in bacterial growth and metabolism, playing a vital role in various cellular processes [27]. The robust iron sequestration capability of *Kluyvera* may account for the reduction in *E. coli* O157:H7 observed in this study. However, experimental confirmation is necessary to validate this hypothesis.

Compost is rich in the nutrients, and studies have shown the growth of foodborne pathogens in compost under favorable conditions [12,21]. Data from Table 1 showed CE reduced slightly more *E. coli* O157:H7 population at lower temperatures (22 °C) in compost with higher moisture contents (30 and 40%). Being a mesophile, *E. coli* O157:H7 is expected to grow faster in higher moisture compost at 30 °C than at 22 °C. In contrast, the CE strains isolated from the finished compost grow better at room temperature. Due to the high growth rate of CE microorganisms in compost with high MC, it’s unsurprising that more *E. coli* O157:H7 was inactivated at room temperature than at 30 °C.

Even though animal manure-based compost is highly recommended for use as the organic fertilizer or biological soil amendment in agricultural production, inadequately treated or handled compost has been implicated in a few produce-related outbreaks [1,3,28]. It is well-documented that foodborne pathogens, such as *Salmonella* spp., can regrow in composted biosolids and stored biosolids [29]. However, only a few studies have examined the growth potential of pathogens in animal manure-based compost. Kim and Jiang [30] reported that *E. coli* O157:H7, *Salmonella* spp., and *Listeria monocytogenes* were able to grow ca. 2–4 logs in 3 days in compost in a greenhouse setting under different seasons when the population of indigenous microorganisms was low (<3 logs CFU/g) and moisture content at least 30–40%. To evaluate the impact on pathogen growth in compost, our CE treatment trials investigated several factors, such as temperature, compost moisture, and physiological stages of *E. coli* O157:H7, which were considered key factors influencing the fate of enteric bacterial pathogens in the environment [15,30]. In this study, the maximal reduction in *E. coli* O157:H7 was 2 logs under greenhouse conditions, which is similar to our previous study on inhibiting *L. monocytogenes* in compost using CE microorganisms [19]. Up to 2.2 log inhibition of *L. monocytogenes* in both compost extract and compost samples by compost-adapted CE microorganisms was reported, and the inhibition was affected by compost types, nutrient levels, and incubation temperatures. These results suggest the effectiveness of applying CE microorganisms to control foodborne pathogens in the finished compost.

Due to the temperature gradients formed across the composting heaps or piles, some populations of bacterial pathogens may be heat-shocked and survive the composting process by adapting to sublethal temperatures. Singh et al. [15] reported that heat-shocked *E. coli* O157:H7, *Salmonella* spp., and *L. monocytogenes* extended survival at lethal temperatures (50–60 °C) following heat shock at 47.5 °C for 1 h. Besides developing heat resistance, heat-shock response can also induce cross-resistance to other stressors, including competition from other microorganisms [31]. In this study, it appears that the heat-shocked *E. coli* O157:H7 became more resistant to CE treatment than not heat-shocked pathogen in compost. A possible explanation is that the changes induced by newly expressed heat-shock genes could influence interactions of heat-shocked *E. coli* O157:H7 with other microorganisms [32]. These interactions may affect adhesion, biofilm formation, nutrient utilization, or susceptibility to antimicrobial compounds produced by competing microorganisms. Further study is needed to understand this cross-resistance mechanism.

As stated by Mead [11], factors unique to the field conditions affecting the efficacy of CE treatment should be evaluated. In this study, the reduction in *E. coli* O157:H7 in compost with similar moisture levels and temperatures by CE treatment was noticeably less under greenhouse conditions compared to laboratory conditions. Unlike the controlled environment for laboratory-based studies, pathogen persistence in the greenhouse environment is exposed to various stresses, such as fluctuations in temperature and relative humidity, UV exposure, unsteady airflow, and others.

Based on our research findings, a cocktail of CE microorganisms should be applied a few days prior to the use of the finished compost, preferably in the colder seasons. The advantage of treating the finished compost with the compost-isolated CE microorganisms is that (i) these CE microorganisms are adapted to the compost environment, thus ensuring their survival; (ii) this biological control method ensures microbiological safety to the compost; and (iii) avoid major changes in compost physicochemical and microbiological properties.

## 5. Conclusions

Our results demonstrated that up to 99% population of *E. coli* O157:H7 cells, resulting from cross-contamination, can be effectively reduced within 2 days during colder seasons (winter and fall) by CE microorganisms (such as *Brevibacillus*, *Bacillus*, *Pseudomonas*, *Kluyvera* and so on) in the finished dairy compost with at least 30% moisture. For those heat-adapted *E. coli* O157:H7 cells surviving the thermophilic composting process, the inhibitory effects from CE became significant only after 8~12 days, suggesting the cross-resistance of the heat-adapted *E. coli* O157:H7 population. Both higher moisture content in the compost and cold seasons enhanced the activity of CE microorganisms against *E. coli* O157:H7. These results indicate that some indigenous compost microflora can be an efficient tool to control foodborne pathogens in finished compost and reduce the potential for soil and crop contamination. However, factors such as the physiological state of the bacteria, the environmental conditions, and compost moisture levels should be considered. Furthermore, those CE strains should be further characterized to ensure the safety of applying these biological control agents. Based on the results of this study, to prevent pathogen growth in finished compost due to cross-contamination, a cocktail of strains of competitive exclusion microorganisms can be applied a few days prior to the use of the finished compost, preferably in the colder seasons.

## Figures and Tables

**Table 1:** *E. coli* O157:H7 growth reduction in compost of different moisture levels in presence of CE cultures.

	*E. coli* O157:H7 Growth Reduction (log CFU/g) in Compost with Moisture Content (%) of		
Temperature (°C)	20	30	40
22	1.05 *	2.59	3.36
30	2.12	2.25	2.57

* Growth reduction (log CFU/g) of *E. coli* O157:H7 within 3 days in the compost treated with ca. 4 log CFU/g CE cultures as compared with control compost.

**Table 2:** Effect of CE cultures on *E. coli* O157:H7 reduction in the finished dairy compost with different moisture levels under greenhouse conditions.

Season	Moisture (%)	Time (d) Required to Have Difference (*p* < 0.05) in Pathogen Reduction between Treatment and Control	
		Approach #1 *	Approach #2
Summer	40	8	8
	30	8	8
	20	8	12
Fall	40	2	12
	30	2	12
	20	16	16
Winter	40	2	8
	30	2	12
	20	16	16

* Approach #1: CE cocktail was inoculated to the compost 24 h prior to the inoculation of *E. coli* O157:H7 cocktail; Approach #2: heat-adapted *E. coli* O157:H7 was inoculated to the compost 24 h prior to the inoculation of CE cocktail.

## Data Availability

All data generated during this study are included in the published article.

## Supplementary Materials

The following supporting information can be downloaded at https://www.mdpi.com/article/10.3390/pathogens13050361/s1. Table S1: Growth inhibitory activity of ten potential competitive exclusion microorganisms against *E. coli* O157:H7 in nutrient broth and compost under laboratory conditions. Table S2: *E. coli* O157:H7 growth in compost with different moisture levels in the absence and presence of CE cultures at 22 and 30 °C.
