# Supplementary material for: Biological Control of Escherichia coli O157:H7 in Dairy Manure-Based Compost Using Competitive Exclusion Microorganisms
Source: Pathogens. 2024 Apr 27;13(5):361. doi: 10.3390/pathogens13050361 (PMC11124295; doi:10.3390/pathogens13050361)
Supplement: Supplementary file 1 [file pathogens-13-00361-s001.zip › pathogens-2973159-supplementary.pdf]

Table S1. Growth inhibitory activity of ten potential competitive exclusion microorganisms against *E. coli* O157:H7 in nutrient broth and compost under laboratory conditions

**Isolate identification based on 16S rRNA**	***E. coli* O157:H7 log reduction/ml or g**	
	Liquid (TYP)	Compost (stored in laboratory conditions)
Comamonadaceae (unclassified)	2.45±0.15	2.14 ±0.25
Enterobacteriaceae (unclassified)	1.63±0.47	1.06 ±0.33
Comamonas (genus)	1.38 ±0.26	1.44 ±0.28
Kluyvera (genus)	3.92 ±0.38	3.72 ±0.41
Raoultella (genus)	1.89 ±0.19	1.77 ±0.32
Citrobacter spp.	1.13 ±0.29	1.15 ±0.47
Enterobacter #1(genus)	1.75 ±0.34	1.44 ±0.35
Comamonas testosteroni CNB-2	1.75 ±0.17	0.89 ±0.05
Nannocystaceae (family) uncultured *proteobacterium*	1.97 ±0.28	2.36 ±0.44
Enterobacter #2(genus)	2.15 ±0.51	1.93 ±0.27

Table S2. *E. coli* O157:H7growth in compost with different moisture levels in the absence and presence of CE cultures at 22 and 30°C

Temp (°C)	Moisture (%)	CE	log CFU *E. coli* O157/g on days of incubation			
			0	1	2	3
22	20	C	**A***2.30 ± 0.00**a****	**B**4.69 ± 0.01**a**	**B**4.52 ± 0.00**a**	**B**5.00 ± 0.10**a**
		Trt	**A**2.30 ± 0.00**a**	**A**3.64 ± 0.09**a**	**A**3.55 ± 0.17**a**	**A**3.95 ± 0.19**a**
	30	C	**A**2.30 ± 0.00**a**	**B**7.13 ± 0.17**c**	**B**8.43± 0.04**b**	**B**8.46 ± 0.03**b**
		Trt	**A**2.30 ± 0.00**a**	**A**5.40 ± 0.04**b**	**A**5.73± 0.11**c**	**A**5.87 ± 0.09**c**
	40	C	**A**2.30 ± 0.00**a**	**B**6.67 ± 0.17**b**	**B**8.52 ± 0.00**b**	**B**8.50 ± 0.07**b**
		Trt	**A**2.30 ± 0.00**a**	**A**5.46 ± 0.07**b**	**A**5.15 ± 0.11**b**	**A**5.14 ± 0.14**b**
30	20	C	**A**2.30 ± 0.00**a**	**B**5.98 ± 0.09**a**	**B**6.84 ± 0.02**a**	**B**6.54 ± 0.10**a**
		Trt	**A**2.30 ± 0.00**a**	**A**4.42 ± 0.19**a**	**A**4.50 ± 0.02**a**	**A**4.42 ± 0.06**a**
	30	C	**A**2.30 ± 0.00**a**	**B**8.28 ± 0.09**b**	**B**8.43 ± 0.03**b**	**B**8.50 ± 0.09**b**
		Trt	**A**2.30 ± 0.00**a**	**A**5.90 ± 0.07**b**	**A**5.90 ± 0.12**b**	**A**6.25 ± 0.11**c**
	40	C	**A**2.30 ± 0.00**a**	**B**8.72 ± 0.24**c**	**B**8.52 ± 0.00**b**	**B**8.48 ± 0.05**b**
		Trt	**A**2.30 ± 0.00**a**	**A**6.29 ± 0.18**c**	**A**6.17 ± 0.03**b**	**A**5.91 ± 0.04**b**

*Means with different uppercase letter in a column at the same temperature and moisture content are significantly different ($P < 0.05$).

**Means with different lowercase letter in a column at the same temperature and same treatment are significantly different ($P < 0.05$).
